# Compared to NIPPV, HFNC is more dangerous regarding aerosol dispersion and contamination of healthcare personnel: we are not sure

**DOI:** 10.1186/s13054-020-03184-y

**Published:** 2020-08-04

**Authors:** Patrick M. Honore, Leonel Barreto Gutierrez, Luc Kugener, Sebastien Redant, Rachid Attou, Andrea Gallerani, David De Bels

**Affiliations:** ICU Department, Centre Hospitalier Universitaire Brugmann-Brugmann University Hospital, Place Van Gehuchtenplein, 4, 1020 Brussels, Belgium

We read with great interest the recent letter by Remy et al. regarding the use of high-flow nasal cannula (HFNC) in COVID-19 patients [1]. After reading the surviving sepsis campaign guidelines on the management of critically ill adults with coronavirus disease 2019 (COVID-19) [2], we initially shared the concern of Remy et al. regarding the recommendation to use HFNC over non-invasive positive pressure ventilation (NIPPV). However, after extensively reading the available literature, we have changed our perspective. The risk of aerosol formation and dispersion for NIPPV systems are variable depending on setting parameters and model/mask type [3]. Viral filters can be attached to the exhalation line on most of the newer models [3]. High fidelity human model studies have demonstrated that exhaled air disperses to 40 cm with the nasal cannula and to 64 cm at 10 cm H_2_O inspiratory air pressure with a bilevel pressure airway pressure (BiPAP) mask [3]. That distance increased to 85 cm and > 95 cm at 18 cm H_2_O depending on the style of the mask used [3]. In comparison, another mannequin study of HFNC demonstrated that even at the highest setting of 60 L/min, exhaled air dispersion was 17 cm in a healthy lung scenario and only 4.8 cm in a severely diseased lung scenario [3, 4]. In other words, in terms of air dispersion and healthcare contamination, HFNC may be less dangerous. This data suggests that NIPPV should be considered cautiously. Closed-circuit systems with appropriate filters in place are important, as are well-fitting masks and the absence of facial hair on the patient, allowing for tight seals [3]. Helmet BiPAP with an air cushion in place around the neck is safe and should be used if available [3]. All other forms of NIPPV have been associated with higher dispersal distances than high flow oxygen systems and concern for increased risk of nosocomial and healthcare provider infection [3].

## Authors’ response

Kenneth E. Remy^1,2,3^ and Philip A. Verhoef^4,5^

^1^Washington University School of Medicine, Department of Internal Medicine, St. Louis, MO.

^2^Washington University School of Medicine, Department of Anesthesiology, Division of Critical Care Medicine, St. Louis, MO.

^3^Washington University School of Medicine, Department of Pediatrics, Division of Critical Care Medicine, St. Louis, MO.

^4^University of Hawaii-Manoa, Department of Internal Medicine, Manoa, HI.

^5^Kaiser Permanente Hawaii, Honolulu, HI

We thank Honore et al. for their interest in our letter regarding the safety associated with the use of high-flow nasal cannula (HFNC) compared to noninvasive positive pressure ventilation (NIPPV) in COVID-19 patients [5]. We have also kept pace with the ongoing literature, and undeniable from 3 months ago, the landscape for understanding COVID-19 has dramatically changed. However, since the pandemic started, many institutions including those in the New York City region have successfully used NIPPV to avoid intubation and without significantly reported transmission of the aerosolized virus [6]. Initial recommendations by major societies were to limit the use of HFNC up to 15 L/min and then intubate without the use of NIPPV to minimize risk to health care workers by reducing possible viral particle aerosolization [7]. Unfortunately, these recommendations lacked specific evidence. Recent societies in the UK and Italy have moved away from the use of HFNC to NIPPV because of a lack of demonstrated efficacy with HFNC, the high oxygen use, and demonstration that in patients that failed HFNC secondary to hypoxemia; these patients were rescued with NIPPV avoiding invasive ventilation [8, 9].

As the authors point out, although the risk of transmission of bacterial transmission with HFNC is low, the risk of viral spread in live patients (rather than inanimate models) has not been studied. In fact, a number of healthy volunteer studies have been performed showing changes and increases in droplet dispersion with increasing flow rates with HFNC through the use of a smoke-laser illumination technique on a human patient simulators [8, 9]. Likewise, NIPPV, when delivered using a tight-fitting mask, dual limb circuitry with a filter on the expiratory limb on a critical care ventilator may demonstrate decreased dispersion and be very safe [9].

Presently, there is a paucity of live COVID-19 patient studies evaluating the aerosolization of the virus in a patient that changes position (moves from supine to prone, or from standing to sitting), generates differing minute ventilations, uses mouth versus nasal breathing, and similar relevant factors. Unfortunately, studies from Europe and China have demonstrated a close to 50% failure rate of HFNC and need for mechanical ventilation [9].

So where does this leave us? Adopting non-prior to COVID-19 standard of care practices to patients with COVID-19 will likely going to lead to negative consequences. If NIPPV is required rather than carbon dioxide “washout,” than NIPPV should be initiated, particularly if a patient has a history of obstructive sleep apnea or asthma. The real consideration is not whether NIPPV or HFNC can aerosolize viral particles but whether or not this is a clinically meaningful aerosolization posing a significant infection risk, and how this risk compares with providing the appropriate PPV vs. simple, flow-based oxygenation treatment [8]. Additionally, many studies that have evaluated aerosol-generating procedures including the use of HFNC or NIPPV have consistently maintained that manipulation of the nasal prongs, NIPPV mask, or coughing pose the largest risk for transmission to health workers instead of sustained, uninterrupted therapy. With appropriate health care worker personal protective equipment, the most appropriate consideration in managing patients with COVID-19 should be no longer which one may hypothetically increase aerosolization of virus particles, but rather which therapy will improve patient respiratory distress and avoidance of prolonged hospitalization from mechanical ventilation. Although NIPPV seemingly has benefit compared to HFNC in this arena, the data for this and aerosolization remain largely elusive.

## Data Availability

Not applicable.

## Abbreviations

HFNC: High-flow nasal cannula
NIPPV: Non-invasive positive pressure ventilation
BiPAP: Bilevel pressure airway pressure

## References

[1] Remy KE, Lin JC, Verhoef PA (2020). High-flow nasal cannula may be no safer than non-invasive positive pressure ventilation for COVID-19 patients. Crit Care.

[2] Alhazzani W, Moller MH, Arabi YM, et al. Surviving sepsis campaign: guidelines on the management of critically ill adults with coronavirus disease 2019 (COVID-19). Crit Care Med Released on March 20. PMID: 32224769. 10.1097/CCM.0000000000004363. 10.1097/CCM.0000000000004363 PMC7176264 32224769

[3] Whittle JS, Pavlov I, Sacchetti AD, Atwood C, Rosenberg MS. Respiratory support for adult patients with COVID-19. J Am Coll Emerg Physicians Open 2020;10.1002/emp2.12071. doi: 10.1002/emp2.12071. Online ahead of print. 10.1002/emp2.12071 PMC7228246 32427171

[4] Hui DS, Chow BK, Lo T, et al. Exhaled air dispersion during high-flow nasal cannula therapy versus CPAP via different masks. Eur Respir J 2019;53(4). doi: 10.1183/13993003.02339-2018. 10.1183/13993003.02339-2018 30705129

[5] Remy KE, Lin JC, Verhoef PA (2020). High-flow nasal cannula may be no safer than non-invasive positive pressure ventilation for COVID-19 patients. Crit Care.

[6] Richardson S, Hirsch JS, Narasimhan M, et al. Presenting characteristics, comorbidities, and outcomes among 5700 patients hospitalized with COVID-19 in the New York city area. JAMA. 2020;323(20):2052–9. 10.1001/jama.2020.6775. 10.1001/jama.2020.6775 PMC7177629 32320003

[7] Alhazzani W, Møller MH, Arabi YM, et al. Surviving sepsis campaign: guidelines on the management of critically ill adults with Coronavirus disease 2019 (COVID-19). Crit Care Med. 2020;48(6):e440–69. 10.1097/CCM.0000000000004363. 10.1097/CCM.0000000000004363 PMC7176264 32224769

[8] Lyons C, Callaghan M. The use of high-flow nasal oxygen in COVID-19. Anaesthesia. 2020;75(7):843–7. 10.1111/anae.15073. 10.1111/anae.15073 32246843

[9] Carter CA, Aedy H, Notter J. Covid-19 disease: non-invasive ventilation and high frequency nasal oxygenation. Clin Integr Care. 2020;1:100006. 10.1016/j.intcar.2020.100006.

